# Supplementary material for: A fault-intrusion-tolerant system and deadline-aware algorithm for scheduling scientific workflow in the cloud
Source: PeerJ Comput Sci. 2021 Nov 2;7:e747. doi: 10.7717/peerj-cs.747 (PMC8576554; doi:10.7717/peerj-cs.747)
Supplement: Supplemental Information 6 [file peerj-cs-07-747-s006.zip › Myproject1.1/docs/org/cloudbus/cloudsim/VmSchedulerTimeShared.html]

VmSchedulerTimeShared


JavaScript is disabled on your browser.


- Overview
- Package
- Class
- Use
- Tree
- Deprecated
- Index
- Help

- Prev Class
- Next Class

- Frames
- No Frames

- All Classes

- Summary:
- Nested |
- Field |
- Constr |
- Method

- Detail:
- Field |
- Constr |
- Method


org.cloudbus.cloudsim

## Class VmSchedulerTimeShared

- java.lang.Object
- - org.cloudbus.cloudsim.VmScheduler
  - - org.cloudbus.cloudsim.VmSchedulerTimeShared

- Direct Known Subclasses:
  :   VmSchedulerTimeSharedOverSubscription

  ---

    

  ```
  public class VmSchedulerTimeShared
  extends VmScheduler
  ```

  VmSchedulerTimeShared is a VMM allocation policy that allocates one or more Pe to a VM, and
  allows sharing of PEs by multiple VMs. This class also implements 10% performance degration due
  to VM migration. This scheduler does not support over-subscription.

  Since:
  :   CloudSim Toolkit 1.0

- - ### Constructor Summary

    Constructors

    Constructor and Description

    `VmSchedulerTimeShared(java.util.List<? extends Pe> pelist)`

    Instantiates a new vm scheduler time shared.
  - ### Method Summary

    Methods

    | Modifier and Type | Method and Description |
    | --- | --- |
    | `protected boolean` | `allocatePesForVm(java.lang.String vmUid, java.util.List<java.lang.Double> mipsShareRequested)` Allocate pes for vm. |
    | `boolean` | `allocatePesForVm(Vm vm, java.util.List<java.lang.Double> mipsShareRequested)` Allocates PEs for a VM. |
    | `void` | `deallocatePesForAllVms()` Releases PEs allocated to all the VMs. |
    | `void` | `deallocatePesForVm(Vm vm)` Releases PEs allocated to a VM. |
    | `double` | `getMaxAvailableMips()` Returns maximum available MIPS among all the PEs. |
    | `protected java.util.Map<java.lang.String,java.util.List<java.lang.Double>>` | `getMipsMapRequested()` Gets the mips map requested. |
    | `protected int` | `getPesInUse()` Gets the pes in use. |
    | `protected void` | `setMipsMapRequested(java.util.Map<java.lang.String,java.util.List<java.lang.Double>> mipsMapRequested)` Sets the mips map requested. |
    | `protected void` | `setPesInUse(int pesInUse)` Sets the pes in use. |
    | `protected void` | `updatePeProvisioning()` Update allocation of VMs on PEs. |

    - ### Methods inherited from class org.cloudbus.cloudsim.VmScheduler

      `getAllocatedMipsForVm, getAvailableMips, getMipsMap, getPeCapacity, getPeList, getPeMap, getPesAllocatedForVM, getTotalAllocatedMipsForVm, getVmsMigratingIn, getVmsMigratingOut, setAvailableMips, setMipsMap, setPeList, setPeMap, setVmsMigratingIn, setVmsMigratingOut`
    - ### Methods inherited from class java.lang.Object

      `clone, equals, finalize, getClass, hashCode, notify, notifyAll, toString, wait, wait, wait`

- - ### Constructor Detail


    - #### VmSchedulerTimeShared

      ```
      public VmSchedulerTimeShared(java.util.List<? extends Pe> pelist)
      ```

      Instantiates a new vm scheduler time shared.

      Parameters:
      :   `pelist` - the pelist
  - ### Method Detail


    - #### allocatePesForVm

      ```
      public boolean allocatePesForVm(Vm vm,
                             java.util.List<java.lang.Double> mipsShareRequested)
      ```

      **Description copied from class: `VmScheduler`**

      Allocates PEs for a VM.

      **Specified by:**
      :   `allocatePesForVm` in class `VmScheduler`

      Parameters:
      :   `vm` - the vm
      :   `mipsShareRequested` - the mips share

      Returns:
      :   $true if this policy allows a new VM in the host, $false otherwise


    - #### allocatePesForVm

      ```
      protected boolean allocatePesForVm(java.lang.String vmUid,
                             java.util.List<java.lang.Double> mipsShareRequested)
      ```

      Allocate pes for vm.

      Parameters:
      :   `vmUid` - the vm uid
      :   `mipsShareRequested` - the mips share requested

      Returns:
      :   true, if successful


    - #### updatePeProvisioning

      ```
      protected void updatePeProvisioning()
      ```

      Update allocation of VMs on PEs.


    - #### deallocatePesForVm

      ```
      public void deallocatePesForVm(Vm vm)
      ```

      **Description copied from class: `VmScheduler`**

      Releases PEs allocated to a VM.

      **Specified by:**
      :   `deallocatePesForVm` in class `VmScheduler`

      Parameters:
      :   `vm` - the vm


    - #### deallocatePesForAllVms

      ```
      public void deallocatePesForAllVms()
      ```

      Releases PEs allocated to all the VMs.

      **Overrides:**
      :   `deallocatePesForAllVms` in class `VmScheduler`


    - #### getMaxAvailableMips

      ```
      public double getMaxAvailableMips()
      ```

      Returns maximum available MIPS among all the PEs. For the time shared policy it is just all
      the avaiable MIPS.

      **Overrides:**
      :   `getMaxAvailableMips` in class `VmScheduler`

      Returns:
      :   max mips


    - #### setPesInUse

      ```
      protected void setPesInUse(int pesInUse)
      ```

      Sets the pes in use.

      Parameters:
      :   `pesInUse` - the new pes in use


    - #### getPesInUse

      ```
      protected int getPesInUse()
      ```

      Gets the pes in use.

      Returns:
      :   the pes in use


    - #### getMipsMapRequested

      ```
      protected java.util.Map<java.lang.String,java.util.List<java.lang.Double>> getMipsMapRequested()
      ```

      Gets the mips map requested.

      Returns:
      :   the mips map requested


    - #### setMipsMapRequested

      ```
      protected void setMipsMapRequested(java.util.Map<java.lang.String,java.util.List<java.lang.Double>> mipsMapRequested)
      ```

      Sets the mips map requested.

      Parameters:
      :   `mipsMapRequested` - the mips map requested


- Overview
- Package
- Class
- Use
- Tree
- Deprecated
- Index
- Help

- Prev Class
- Next Class

- Frames
- No Frames

- All Classes

- Summary:
- Nested |
- Field |
- Constr |
- Method

- Detail:
- Field |
- Constr |
- Method
